# The Algicidal Fungus *Trametes versicolor* F21a Eliminating Blue Algae via Genes Encoding Degradation Enzymes and Metabolic Pathways Revealed by Transcriptomic Analysis

**DOI:** 10.3389/fmicb.2018.00826

**Published:** 2018-04-27

**Authors:** Wei Dai, Xiaolin Chen, Xuewen Wang, Zimu Xu, Xueyan Gao, Chaosheng Jiang, Ruining Deng, Guomin Han

**Affiliations:** ^1^School of Life Sciences, Anhui Agricultural University, Hefei, China; ^2^Department of Genetics, University of Georgia, Athens, GA, United States; ^3^School of Resources and Environmental Engineering, Hefei University of Technology, Hefei, China; ^4^The National Engineering Laboratory of Crop Stress Resistance Breeding, Anhui Agricultural University, Hefei, China

**Keywords:** transcriptomic analysis, *Trametes versicolor* F21a, algicidal process, metabolic pathway, degradation enzymes

## Abstract

The molecular mechanism underlying the elimination of algal cells by fungal mycelia has not been fully understood. Here, we applied transcriptomic analysis to investigate the gene expression and regulation at time courses of *Trametes versicolor* F21a during the algicidal process. The obtained results showed that a total of 193, 332, 545, and 742 differentially expressed genes were identified at 0, 6, 12, and 30 h during the algicidal process, respectively. The gene ontology terms were enriched into glucan 1,4-α-glucosidase activity, hydrolase activity, lipase activity, and endopeptidase activity. The KEGG pathways were enriched in degradation and metabolism pathways including Glycolysis/Gluconeogenesis, Pyruvate metabolism, the Biosynthesis of amino acids, etc. The total expression levels of all Carbohydrate-Active enZYmes (CAZyme) genes for the saccharide metabolism were increased by two folds relative to the control. AA5, GH18, GH5, GH79, GH128, and PL8 were the top six significantly up-regulated modules among 43 detected CAZyme modules. Four available homologous decomposition enzymes of other species could partially inhibit the growth of algal cells. The facts suggest that the algicidal mode of *T. versicolor* F21a might be associated with decomposition enzymes and several metabolic pathways. The obtained results provide a new candidate way to control algal bloom by application of decomposition enzymes in the future.

## Introduction

Algae, the food of aquatic animals, play a vital role in water ecosystems (Sigee, 2005). However, outbreaks of cyanobacterial blooms often severely decrease water clarity, deteriorate environments of water bodies, deplete oxygen, damage fisheries, and form a threat to public health (Sigee, 2005; Zeng et al., 2015; Sun et al., 2017). Many approaches have been developed to control algal blooms, including physical, chemical, and biological methods (Sigee, 2005; Zeng et al., 2015). Due to the high cost and safety limitations of current approaches, the development of novel strategies remains necessary. Biological control has been recognized as a cost efficient and ecologically sound method for eliminating the growth of harmful algae compared to chemical and other biological methods (Sigee, 2005).

Compared to studies of algal antagonistic organisms, such as bacteria and viruses, the study and application of fungi for the elimination of algal cells has not received much attention, until recently, when Jia et al. (2010) reported a new way of eliminating living algae with fungi. After that, investigations into the application of fungi for the restoration of algal blooms are growing (Han et al., 2011; Mohamed et al., 2014; Zeng et al., 2015; Shu et al., 2016). The fungus *Trichaptum abietinum* 1302BG could directly eliminate four tested algal species when fungal mycelia were co-cultured with algal cells (Jia et al., 2010). In addition to *T. abietinum* 1302BG, several fungi, e.g., *Lopharia spadicea, Phanerochaete chrysosporium, Trichoderma citrinoviride, Irpex lacteus* T2b, *Trametes versicolor* F21a, and *Bjerkandera adusta* T1 also show similar algicidal ability (Wang et al., 2010; Han et al., 2011; Mohamed et al., 2014; Zeng et al., 2015). Scanning electron microscopic and transmission electron microscopic observations showed that fungal mycelia first directly contact with algal cells, and then damage the algal cells (Jia et al., 2010). Zeng et al. (2015) reported that the membranes of the algal cells and the pyrrole ring of chlorophyll-a can be damaged by *P. chrysosporium*. Du et al. (2015) suggested that cellulase, β-glucosidase, protease, and laccase of *T. versicolor* F21a are potential extracellular enzymes which can be involved in eliminating *Microcystis* spp. cells during the early stages (0 to 24 h), while β-glucosidase, protease, laccase, and manganese peroxidase could be involved in the late stages (24 to 60 h). In a recent study, our group detected 30 fungal enzymes with endo- or exo-glycosidase activities such as β-1,3-glucanase, α-galactosidase, α-glucosidase, alginate lyase, chondroitin lyase, peptidase, exonuclease, and manganese peroxidase, which should be involved in the algicidal process of fungus *T. versicolor* F21a (Gao et al., 2017). However, gene ontology (GO) terms or KEGG pathways related to degradation were not directly observed from the proteomic data, which might be due to the limitations of the currently available proteomics techniques. 14,296 protein coding sequences were predicted in the reference genome of *T. versicolor* FP-101664 SS1 (Floudas et al., 2012); nevertheless, only ~1/4 of the proteins including the degradation enzymes could be detected via the proteomics analysis. Although a few extracellular fungal enzymes were detected in the algicidal process of *T. versicolor* F21a, we still do not know how many types of decomposition genes and metabolic pathways are involved and lack of convincing evidence whether the degradation enzymes play vital roles as proposed in previous studies.

In this study, RNA-Seq based transcriptomic technique was applied to examine the gene expression and regulation at time courses during the algicidal process. Furthermore, bioinformatic analysis was used to investigate all metabolic pathways and highly up-regulated genes for decomposition in the algicidal process of *T. versicolor* F21a. Finally, the results of bioinformatics analysis were further verified by experiments. The results of this study can enrich our understanding of the molecular interaction between fungi and algae.

## Materials and methods

### Fungal and algal strains

The previously isolated fungus *T. versicolor* F21a from the Zijinshan Mountain was used for this investigation (Han et al., 2011). Algal strain *Microcystis aeruginosa* PCC7806 was provided by the Institute of Hydrobiology of the Chinese Academy of Sciences (Wuhan, China).

### Co-cultivation of the fungal mycelia and algal cells

The algal strain was cultivated at 25°C under 12 h light and 12 h dark cycles with ~90 μmol m^−2^ s^−1^ of photons in BG-11 medium (Jia et al., 2010). Round fungal mycelium (with 7 mm in diameter) was inoculated onto a 9 cm plate containing 15 mL of potato liquid medium under static conditions for 5 days. Then the fungal mycelia were picked up and transferred into 250 mL Erlenmeyer flasks containing 100 mL of algal solution or medium. The co-cultures were incubated at 25°C, 90 μmol photons m^−2^ s^−1^, and 120 rpm to observe the interaction and to investigate differentially expressed fungal genes. Total chlorophyll-a was measured according to the Standard Methods for the Examination of Water and Wastewater (1998).

### Transcriptome sequencing

Samples were collected from co-cultivates at 0, 6, 12, and 30 h. Two biological replicates of each treatment were used for RNA-Seq. The total RNA was extracted from each sample with Trizol reagent according to manufacturer's instructions (Takara, Dalian, China). Then, the crude RNA was digested via 10 U DNase I (TaKaRa, Japan) at 37°C for 30 min. mRNA was isolated from the crude RNA via the Dynabeads® Oligo (dT) 25 (Life, America) according to manufacturer's instructions. 100 ng mRNA of each sample was used to construct the sequencing library with NEBNext® UltraTM RNA Library Prep Kit (NEB, America). Paired-end sequencing of cDNA fragments (~300 bp) were conducted at the Illumina HiSeq 4000 platform by BGI-shenzhen, China.

### Bioinformatics analyses

The quality of 150-bp reads was assessed via the software FASTQC (http://www.bioinformatics.babraham.ac.uk/projects/fastqc/). The paired-end raw reads from RNA-Seq were trimmed, thus removing low quality base-calls (*Q* < 30) and adaptor sequences with pipeline Fastq_clean (v2.0) (Mi et al., 2014). The cleaned reads were mapped to the reference genome of *T. versicolor* via STAR (v2.5.3a) (Dobin et al., 2013; Dobin and Gingeras, 2015). The differentially expressed genes (DEGs) in fungi were calculated according to the FPKM method via Cuffdiff (v2.2.1) using default parameters (*p* < 0.05, fold of change ≥ 2) (Trapnell et al., 2012). Gene functions were annotated via the BLAST pipeline against the references of the protein-encoding sequence from the Nr of GenBank, GO (Ashburner et al., 2000), and Kyoto Encyclopedia of Genes and Genomes (KEGG) (Kanehisa and Goto, 2000). Fisher's exact test was used to obtain enriched functional terms (*p* < 0.05). Genes encoding lignocellulose-active enzymes were further annotated via dbCAN (Lepoivre et al., 2012; Yin et al., 2012).

### Verification of DEG expression via real-time quantitative PCR

Real-time quantitative PCR was used to verify the gene expression level calculated from RNA-Seq data. A few arbitrarily selected lignocellulose-active enzyme genes were used in this study, and the β-actin gene of *T versicolor* F21a was used as endogenous control. The 20 μL reaction system consisted of 120–150 ng cDNA, gene-specific primers (0.5 μL, 10 μmol L^−1^) (Supplementary Table 1) and 5 × SYBR Green Master Mix (10 μL). The real-time quantitative PCR program was set as follows: 95°C for 10 min, followed by 40 cycles of 95°C for 15 s, 60°C for 20 s, and 72°C for 30 s. Relative expression levels were calculated using the 2^−ΔΔ^CT method (Livak and Schmittgen, 2001). Three biological replicates were used for the Real-time quantitative PCR.

### Effects of commercial decomposition enzymes on algal cells

Available commercial decomposition enzymes were purchased from Hefei Bomei Biotechnology CO., LTD, China. Cellulase (0, 100, and 500 U), β-glucanase (0, 5,000, and 9,000 U), Trypsase (0, 3,000, and 5,000 U), Pepsin (0, 5,000, and 10,000 U) were prepared according to the instructions and added to 4 mL algal solutions to assess the effects of decomposition on algal cells, respectively.

## Results

### Dynamics of fungus-alga interactions

**Figure 1:**
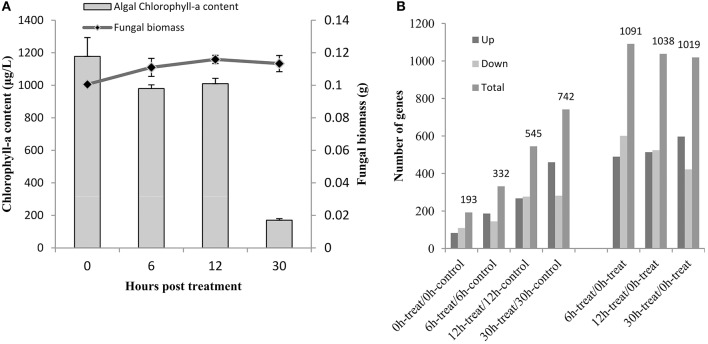
Chlorophyll-a content and numbers of fungal DEGs during the algicidal process. **(A)** Changes of chlorophyll-a content during the algicidal process. **(B)** The number of fungal DEGs between different samples; Up, up-regulated DEGs; Down, down-regulated DEGs; Total, total number of DEGs.

The interaction between *T. versicolor* F21a and algal cells was monitored via spectrophotometer. Figure 1A shows that the chlorophyll-a content of algal cells was gradually decreased during the first 12 h, reaching one sixth of the initial content after 30 h. About 85% algal cells were eliminated within 30 h. The biomasses of the fungal mycelia after 30 h co-cultivation were significantly increased by ~12% compared with 0 h pure mycelia (*p* < 0.05).

### RNA-Seq analysis of gene expression in the fungal mycelia during the algicidal process

Electrophoresis of total RNAs extracted from mycelia or co-cultivated mixture of mycelia and algal cells showed that the RNAs were good enough for cDNA library construction (Supplementary Figure 1). The prokaryotic mRNA in blue algae lacks poly A tail, eukaryotic mRNA contains a poly A tail. Thus, fungal mRNA could be distinguished from mRNA of blue algae via oligo(dT) capturing. Sequencing of all samples yielded 66,343,647 raw paired-end 150-bp reads (Supplementary Table 2). The quality of raw reads (SRA accession: SRP127790) showed that an overwhelming majority of the reads had quality scores above Q30 (Supplementary Figure 2). After removing adaptor and unknown sequences and discarding low quality reads, 99.88% of reads remained as clean reads with an average length of ~143 bp, which could be used for mapping onto the reference genome of *T. versicolor* for measuring gene expression level (Supplementary Table 2).

### Identification and validation of DEGs

The genome sequence of the very closely related *T. versicolor* FP-101664 SS1, harboring 14,296 putative protein-coding genes from the Joint Genome Institute, was used as reference genome (Floudas et al., 2012). Supplementary Table 2 shows that more than 65% of paired clean reads with an average length of 272 bp can be uniquely mapped to the reference genome via pipeline STAR. 9,517 predicted fungal genes in total could be detected throughout all samples at the cutoff FPKM > 1 in at least one sample. The number of expressed genes detected via RNA-Seq was twice as that detected by proteomics in our previous study.

Figure 1B shows that a total of 193, 332, 545, and 742 fungal DEGs were identified at 0, 6, 12, and 30 h in mycelia co-cultivated with algal cells compared with pure mycelia, respectively. The number of fungal DEGs was increased with prolonged incubation time. 1091, 1038, and 1019 fungal DEGs were detected at 6, 12, and 30 h in mycelia co-cultivated with algal cells compared with that of 0 h (Figure 1B). Of these, about half of all DEGs were up-regulated (Supplementary Table 3). 207 up-regulated genes and 140 down-regulated genes were previously detected via proteomics (Gao et al., 2017). The number of DEGs detected via RNA-Seq was much higher than that detected by proteomic analysis.

To verify the reliability of DEGs identified via RNA-Seq, the relative expression levels of arbitrarily selected CAZyme genes were further investigated via real-time PCR. The result showed that a similar expression pattern was observed between real-time PCR and transcriptomic analyses (Supplementary Figure 3). This indicates that the relative expression level identified by the transcriptome was reliable and suitable for further analyses.

### Function and enrichment analyses of fungal DEGs

**Figure 2:**
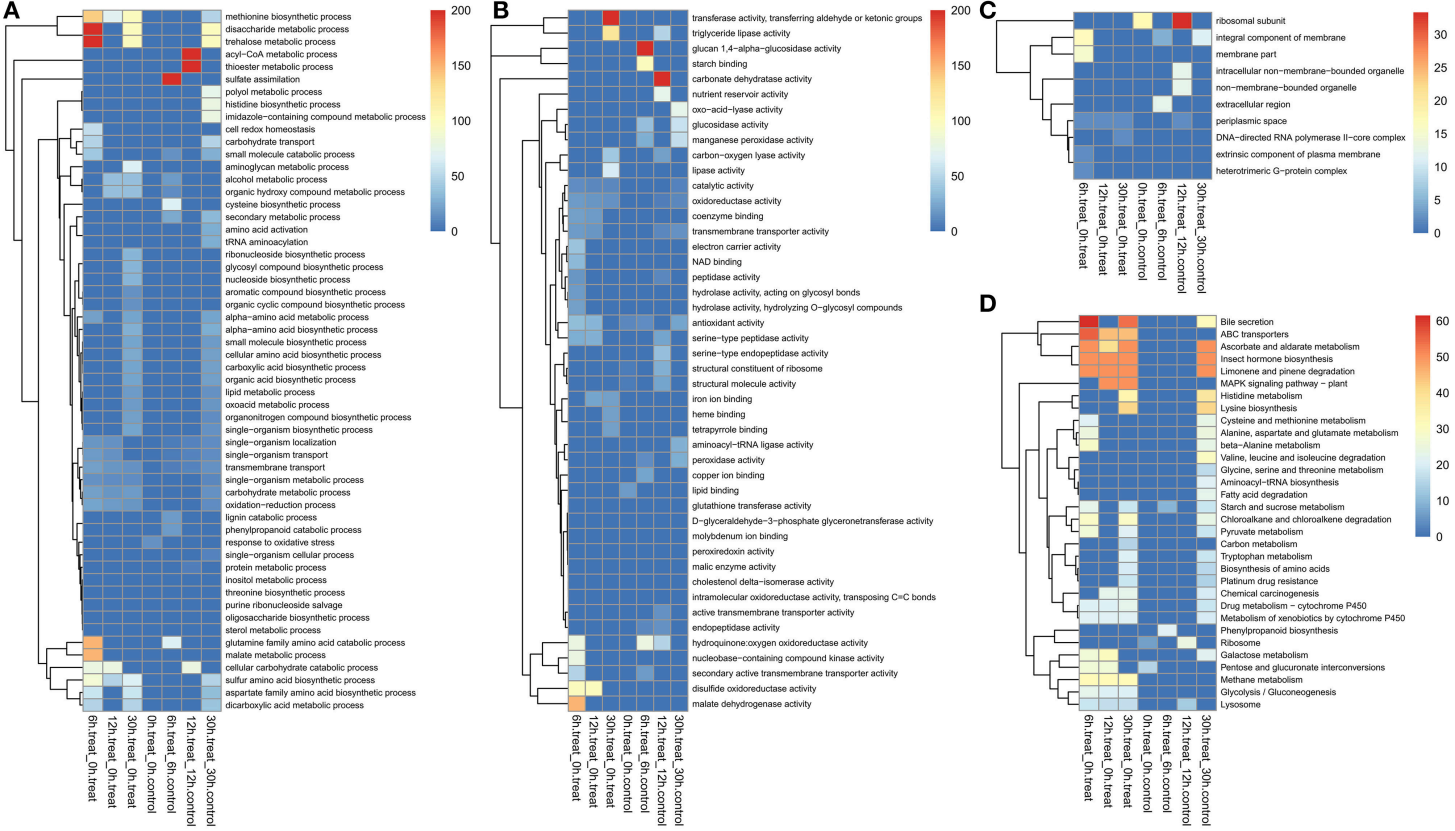
GO and KEGG term enrichments of fungal DEGs during the algicidal process. **(A)** Biological process, **(B)** Molecular function, **(C)** Cellular component, and **(D)** KEGG pathway.

The GO terms of identified DEGs were enriched into many carbohydrate metabolic and transportation related processes in the biological process category, including the trehalose metabolic process, the disaccharide metabolic process, the malate metabolic process, the thioester metabolic process, the acyl-CoA metabolic process, the lipid metabolic process, the transmembrane transport, the organonitrogen compound biosynthetic process, and the protein metabolic process (Figure 2A). The GO terms of identified DEGs were enriched into many degradation related activities in the molecular function category, including glucan 1,4-α-glucosidase activity, carbonate dehydratase activity, oxo-acid-lyase activity, hydrolase activity, lipase activity, endopeptidase activity, and manganese peroxidase activity (Figure 2B). GO terms of identified DEGs were enriched into both the ribosome and the membrane in the cellular component category, including the ribosomal subunit, the intracellular non-membrane-bounded organelle, and the integral component of the membrane extracellular region (Figure 2C).

The KEGG analysis of identified DEGs were enriched into several degradation and metabolic pathways during the early algicidal stages, including Bile secretion, ascorbate and aldarate metabolism, limonene and pinene degradation, cysteine and methionine metabolism, β-Alanine metabolism, starch and sucrose metabolism, pyruvate metabolism, chloroalkane and chloroalkene degradation, the metabolism of xenobiotics by cytochrome P450, pentose and glucuronate interconversions, galactose metabolism, methane metabolism, glycolysis/gluconeogenesis, and the lysosome (Figure 2D). MAPK signaling pathway, chemical carcinogenesis, ribosome, and phenylpropanoid biosynthesis pathway appeared gradually during fungal-alga interaction (Figure 2D). Histidine metabolism, lysine biosynthesis, valine, leucine and isoleucine degradation, glycine, serine, and threonine metabolism, fatty acid degradation, aminoacyl-tRNA biosynthesis, tryptophan metabolism, biosynthesis of amino acids, and carbon metabolism were the main enriched pathways during the late stages (Figure 2D). The majority of the enriched KEGG pathways were consistent with the results of GO analyses.

### Composition and expression of fungal decomposition genes during algicidal process

**Figure 3:**
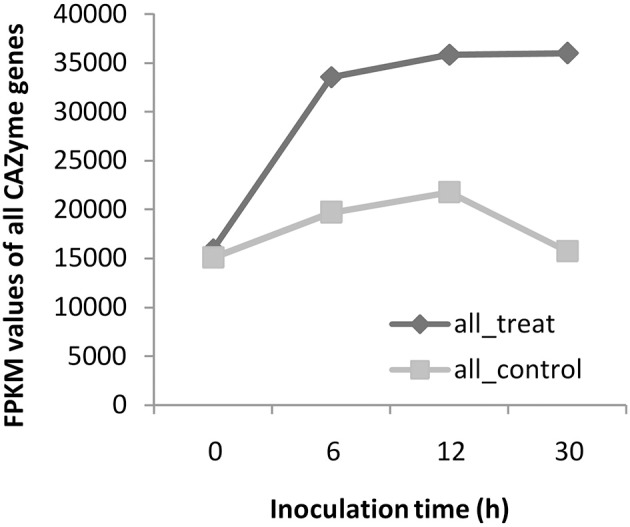
Total expression levels of all CAZyme genes during the algicidal process.

A total of 312 genes with predicted lignocellulose degradation capabilities exist in the fungal genome. Of those, ~70% genes (220 lignocellulose genes) were detected via RNA-Seq, and ~23% (115 lignocellulose genes) were differentially expressed genes (Table 1, Supplementary Table 4, Supplementary Figure 4). The total expression levels of CAZyme genes during the algicidal process were almost twice as that of the control (Figure 3). The majority of detected and differentially expressed lignocellulose genes belong to the Glycoside Hydrolases (GH) family and the Auxiliary Activities (AA) family (Supplementary Figure 4). Only 11 and five DEGs detected via RNA-Seq belong to the Carbohydrate Esterases (CE) family and the Polysaccharide Lyases (PL) family, respectively (Supplementary Figure 4). The number of lignocellulose genes detected via transcriptomic analyses was almost doubled, compared with the 84 lignocellulose genes detected by the previous proteomic analysis.

**Table 1:** The numbers of decomposition enzyme detected by RNA-Seq and our previous proteomic study of *T. versicolor* F21a.

**Enzyme classes**	**CAZyme module**	**No. of decomposition enzymes in genome**	**No. of decomposition enzymes detected by RNA-Seq**	**No. of decomposition enzymes detected by our previous proteomic study**	**No. of decomposition enzymes in DEGs by RNA-Seq**
Auxiliary activities	AA2	27	21	4	11
	AA3	23	13	9	10
	AA5	9	5	3	3
	AA8	2	1	0	
	AA9	18	13	0	3
Carbohydrate esterases	CE1	26	19	14	8
	CE12	2	2	2	1
	CE4	5	3	2	2
	CE8	2	1	0	
Glycoside hydrolases	GH1	2	2	1	1
	GH10	6	5	0	2
	GH115	2	1	1	1
	GH12	5	4	0	2
	GH128	4	3	0	3
	GH13	6	5	3	4
	GH15	4	3	1	2
	GH16	29	20	4	12
	GH17	1	1	1	1
	GH18	20	16	2	9
	GH2	5	4	1	2
	GH27	3	2	1	2
	GH28	9	6	1	4
	GH3	12	7	6	2
	GH30	4	4	0	2
	GH31	5	3	4	2
	GH32	3	3	1	1
	GH35	2	2	1	1
	GH43	3	2	0	
	GH45	1		0	
	GH47	5	3	4	1
	GH5	22	14	8	8
	GH51	2	2	0	1
	GH53	1	1	0	
	GH55	2	2	1	2
	GH6	1	1	0	
	GH7	4	4	0	1
	GH71	5	2	0	1
	GH74	2	1	1	1
	GH78	4	1	0	
	GH79	11	8	1	4
	GH85	1	1	1	
	GH9	1	1	0	
Polysaccharide lyases	PL14	6	6	2	3
	PL15	2		2	
	PL4	1		0	
	PL8	2	2	2	2
	Total	312	220	84	115

**Figure 4:**
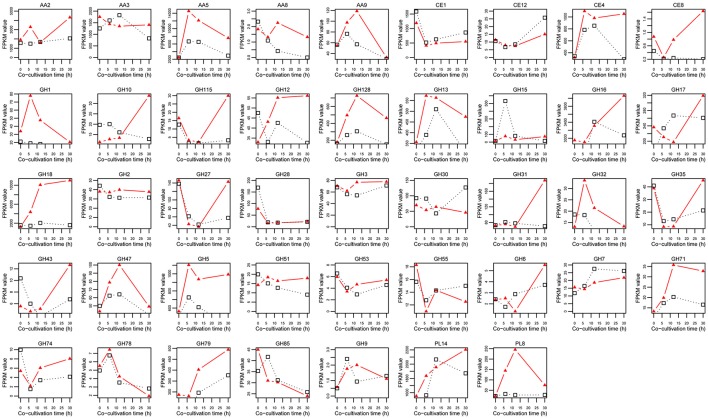
Total expression levels of each CAZyme module during the algicidal process.

**Figure 5:**
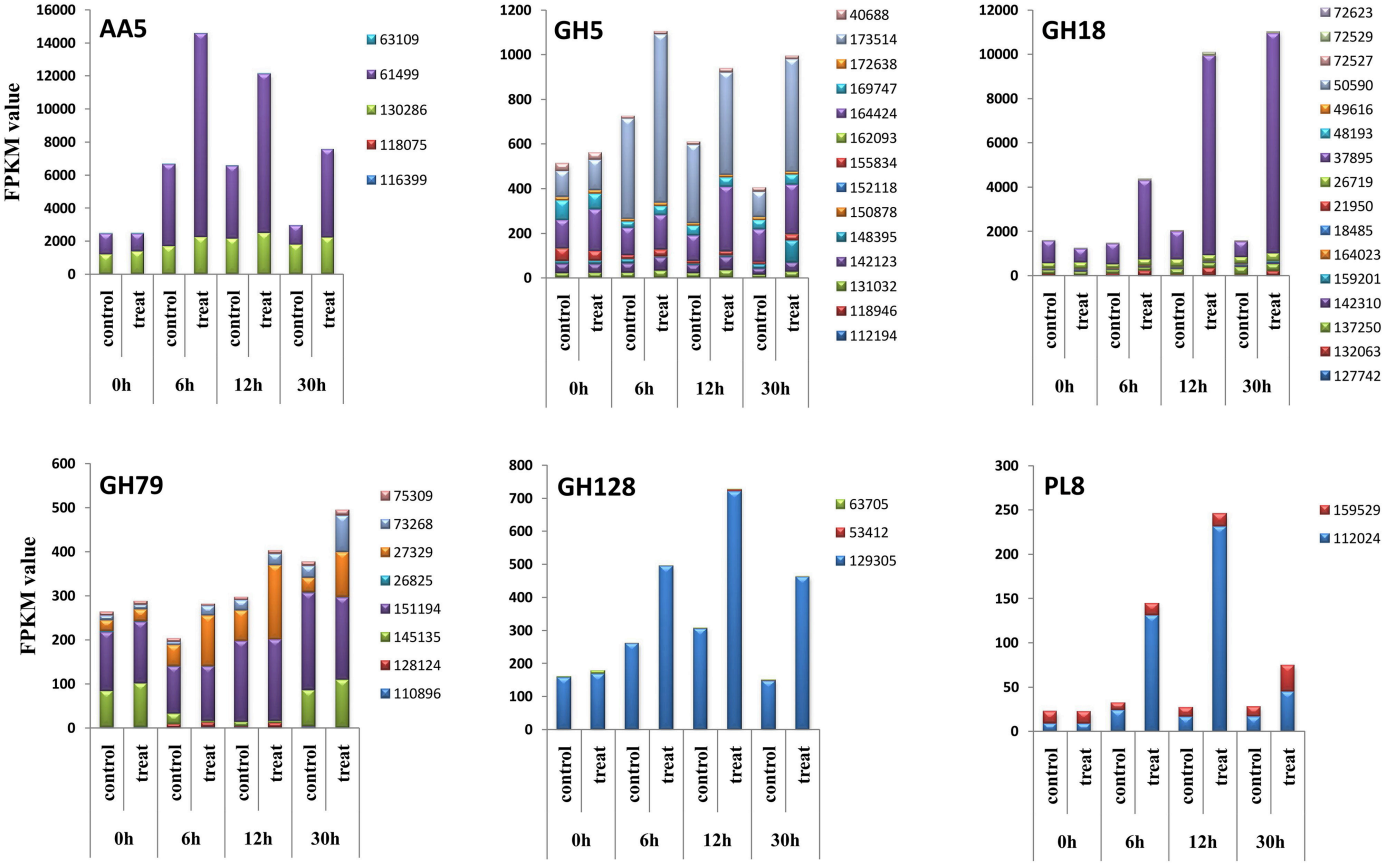
Expression levels of genes in six modules during the algicidal process.

The 312 genes presented above were further divided into 46 modules, 43 of which could be detected via RNA-Seq (Table 1, Supplementary Table 4). Thirty-five modules contained 115 differentially expressed lignocellulose genes (Table 1, Supplementary Table 4). The total expression levels of all genes within the same module showed varied patterns during the algicidal process (Figure 4). Many fungal genes were found to belong to modules, e.g., AA5, AA9, CE4, GH1, GH5, GH13, GH18, GH32, GH47, GH71, GH79, GH128, and PL8, which were rapidly induced when co-cultivated with algal cells. AA5, GH18, GH5, GH79, GH128, and PL8 were the top six accumulated up-regulated modules. In particular, the FPKM values of genes in both the AA5 and the GH18 modules in treated samples were above 10,000, far exceeding the genes in the remaining modules (Figure 4). The expression level of the transcript with ID 37895 was the highest among the 20 members of the GH18 module, while the expression level of the transcript with the ID 61499 was the highest among nine members in the AA5 module (Figure 5). The expression of the transcript with the ID 37895 in GH18 module increased by ~40% from the beginning of treatment to 30 h after treatment, while it decreased by 16% in the control (Supplementary Figure 5). Similarly, the transcript with the ID 173514 in the GH5 module, the transcripts with the IDs 151194 and 37329 in the GH79 module, the transcript with the ID 129305 in the GH128 module, and the transcript with the ID 112024 in the PL8 module accounted for the predominant transcripts in the corresponding modules (Figure 5). This indicates that these genes play vital roles in eliminating different debris of algal cells.

### Inhibitory effects of commercially available decomposition enzymes on algal cells

**Figure 6:**
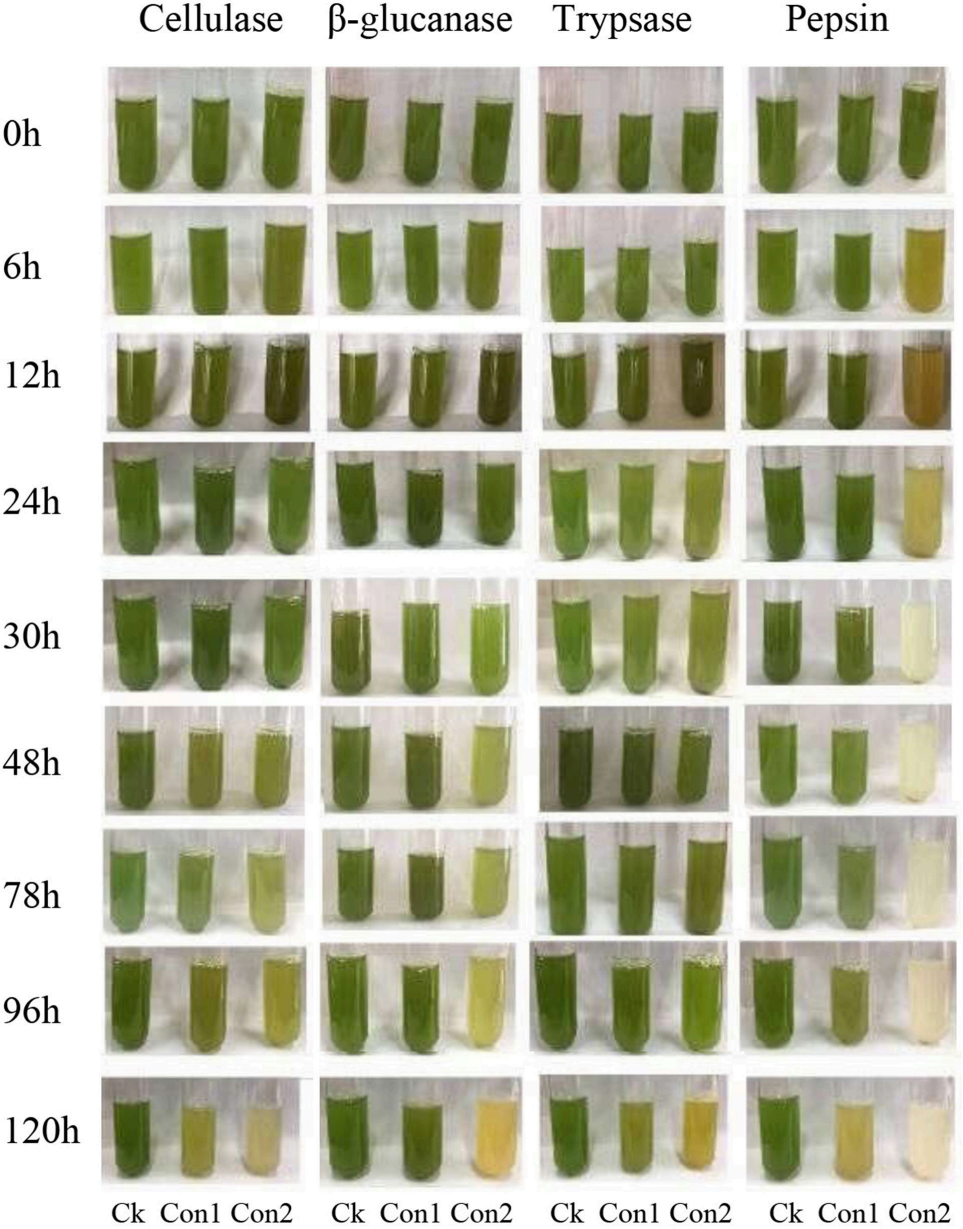
Effects of commercial decomposition enzymes on algal cells. Cellulase (Con1, 100 U; Con2, 500 U), β-glucanase (Con1, 5,000 U; Con2, 9,000 U), trypsase (Con1, 3,000 U; Con2, 5,000 U), and pepsin (Con1, 5,000 U; Con2, 10,000 U).

To test the roles of the decomposition enzymes in the results mined by our bioinformatics analysis, several identified enzymes with endo-glycosidase activities and endopeptidase activity were purchased and applied to test their inhibitory abilities on algal cells. Figure 6 shows that cellulase, β-glucanase, trypsase, and pepsin can partially inhibit the growth of algae at high concentrations compared with the respective control. The green color almost disappeared during 30–120 h in the algal solution when treated with high pepsin concentration.

## Discussion

In this study, ~66% of the predicted fungal genes could be detected during the algicidal process via RNA-Seq. The number of detected genes far exceeded that obtained via proteomic techniques, which was also observed in many other studies (Bai et al., 2015; Li et al., 2017). A previous proteomic study showed that pyruvate metabolism and tricarboxylic acid cycle pathways, carbon metabolism, selenocompound metabolism, sulfur assimilation and metabolism, and several amino acid biosynthesis pathways had been enriched (Gao et al., 2017). The pathways that were enriched in the present study included almost all enriched pathways obtained via proteomic analysis (Figure 2D). The results of GO term enrichment clearly showed that many degradation activities were related to polymeride decomposition and the conversion of small molecules; e.g., glucan 1,4-α-glucosidase activity, lipase activity, and the endopeptidase activity at a molecular function level. The degradation activities related GO terms were directly enriched during the algicidal process, and were first observed by the present study. Glucan 1,4-α-glucosidase (also called amylase) catalyzes the hydrolysis of starch into sugars on α-1,4-glycosidic bonds. A few studies have investigated the utilization of microalgae or algae-based glucose to produce bioethanol and bacterial cellulose (Chen et al., 2015; Uzyol and Sacan, 2017), which has shown a promising future for algal polysaccharides. Lipases can hydrolyze triacylglycerols with long-chain fatty acids, thus releasing free fatty acids, diacylglycerols, monoacylglycerols, and glycerol, which can be used to produce polyunsaturated fatty acids and biodiesel (Mohamed et al., 2014; Morales-Medina et al., 2018). Several studies also reported that endopeptidases often decompose proteins into amino acids. These enzymes might be involved in the decomposition of polysaccharides, lipids, and proteins of algal cells into small molecules. The enriched GO terms at the biological process level, i.e., trehalose metabolic process, disaccharide metabolic process, lipid metabolic process, carbohydrate transport, and protein metabolic process, should be involved in the conversion of small molecules of monosaccharides, disaccharides, fatty acids, and amino acids into other types of compounds and macromolecules which are useful for fungal cells. The result of enriched GO terms implies that the macro molecules of algal cells should be first broken down into small molecules, which were then further used as substrates for subsequent different metabolic pathways. The lysis of algal cells is the first step to eliminate them, and decomposition enzymes play vitally important roles for this process.

Lignocellulose genes are most important for degradation (Yin et al., 2012; Strasser et al., 2015). Here, we identified 70% lignocellulose genes of all CAZymes, which is more than those found in the previous proteomic analysis (Gao et al., 2017). Most of up-regulated lignocellulose-active enzymes previously detected via proteomic analysis were included in the gene list identified in this study (Supplementary Table 4). Thus, overlapped genes would be of high confidence for the degrading roles. Many CAZyme proteins are involved in several different modules, enabling them to target specific or divergent substrates, implying that the elimination of algal cells by *T. versicolor* F21a is a very complex process. Among these modules, GH18, AA5, GH5, GH79, GH128, and PL8 were the top ranked up-regulated modules, which suggest that these modules may be the key for eliminating algal cells.

Eight genes in the module GH18 were significantly up-regulated during the algicidal process (Supplementary Table 4). Of these eight genes, transcript ID 37895 was the highest expressed gene and its annotation showed that it encodes a hydrolase for hydrolyzing o-glycosyl compounds. This implies that transcript ID 37895 should be one of the key enzymes responsible for the breakdown of peptidoglycan of algal cell walls. Furthermore, lysozyme (EC 3.2.1.17), endo-β-N-acetylglucosaminidase (EC 3.2.1.96), and peptidoglycan hydrolase with endo-β-N-acetylglucosaminidase specificity (EC 3.2.1.-) belong to the GH18 module with the capacity to hydrolyze many different types of polymerides. Peptidoglycan and cellulose are cell wall components of cynobacteria and eukaryotic algae, respectively. It has previously been reported that algicidal fungi *T. abietinum* 1302BG, *L. spadicea*, and *P. chrysosporium* can eliminate prokaryotic and eukaryotic algal cells (Jia et al., 2010; Wang et al., 2010; Zeng et al., 2015). *T. versicolor* F21a can also efficiently eliminate eukaryotic algae and algal bloom samples from Taihu Lake, China in our test (unpublished data). Enzymes of the GH18 module might play a central role during the initial algicidal stage by disrupting cynobacterial cell walls. They could possibly also play important roles in decomposing the cell walls of eukaryotic algae.

GH5, GH79, GH128, and PL8 were also significantly up-regulated during the algicidal process. Endo-β-1,4-glucanase (EC 3.2.1.4), β-1,4-cellobiosidase (EC 3.2.1.91), glucan β-1,3-glucosidase (EC 3.2.1.58), endo-β-1,4-xylanase (EC 3.2.1.8), and endo-β-1,4-mannosidase (EC 3.2.1.78) of the GH5 module can decompose cellulose, cellobioside, β-1,3-glucans, xylans, and galactomannans into glucose, xylose, galactose, and mannose (Yin et al., 2012; Blackman et al., 2014; Thomas et al., 2017). Xyloglucanase (EC 3.2.1.151) and endo-1,3-β-glucanase (EC 3.2.1.39) of GH16, β-1,3-glucanase (EC 3.2.1.39) of GH128 can decompose xyloglucans and β-1,3-glucans into xylose and glucose (Yin et al., 2012; Blackman et al., 2014). Hyaluronoglucuronidase (EC 3.2.1.36) of the GH79 module can randomly hydrolyze (1 → 3)-linkages between β-D-glucuronate and N-acetyl-D-glucosamine residues in hyaluronate into hyaluronate oligosaccharides (Yin et al., 2012). Heparanase (EC 3.2.1.166) of the GH79 module can endohydrolyze (1 → 4)-β-D-glycosidic bonds of heparan sulfate chains in heparan sulfate proteoglycan from proteoglycan core proteins and degrade these into small oligosaccharides (Gong et al., 2003). Chondroitin ABC lyase (EC 4.2.2.1) of PL8 and alginate lyase (EC 4.2.2.3) of PL14 can both decompose peptidoglycan and alginate, which expression level was also significantly up-regulated and consistent with the previous proteomics study (Gao et al., 2017). Many peptidase and lipase genes were also significantly up-regulated during the algicidal process, which clearly indicates that the underlying algicidal mechanism is complicated, and suggests that many decomposition genes should participate in the process. Of these genes, transcript ID: 37895 of GH18, 173514 of GH5, and 112024 of PL8 might play key roles in the breakdown of the peptidoglycan of algal cell walls, resulting in the breaking of cells. Many other different types of decomposition enzymes might further degrade the debris of algal cells into small molecules, such as glucose.

It is worth mentioning that the FPKM values of genes in the AA5 module were above 10,000, which far exceeded that in the remaining modules, except for GH18, suggesting that enzymes of AA5 play an important role in eliminating the debris of algal cells (Figure 5). Transcript ID 61499 was the major expressed gene among the nine genes in AA5. The protein of transcript ID 61499 encodes a glyoxal oxidase (EC 1.2.3.15), a H_2_O_2_-generating copper radical oxidase. This enzyme can catalyze the oxidization of simple aldehyde, glyoxal, methylglyoxal, α-hydroxycarbonyl, or α-dicarbonyl compounds as substrates to generate pyruvate and extracellular H_2_O_2_ (Kersten and Kirk, 1987; Vanden Wymelenberg et al., 2006; Yin et al., 2012). Several studies reported that malondialdehyde (MDA) content was greatly increased during the algicidal process (Jia et al., 2010; Wang et al., 2010; Zeng et al., 2015), and then gradually decreased during later stages (Wang et al., 2010). MDA is the final product of membrane lipid decomposition, which reflects the degree of injury of algal cells (Zeng et al., 2015). The high expression level of glyoxal oxidase genes observed in this study could be responsible for the removal of generated MDA during the algicidal process, and for the resulting decline of the MDA content during the later stage. Transcript ID 61499 is likely the key coding sequence for glyoxal oxidase. This may also explain why this fungus has strong inhibitory effects on living algal cells.

As suggested above, enzymes with endo-glycosidase activity and endopeptidase activity should play a key role in the first step to break algal cells. We tested our hypothesis by assaying several available commercial decomposition enzymes that target the components of algal cell walls. GH18 member cellulase and endo-β-glucanase were purified from *Aspergillus niger*, which have been known to cleave the peptidoglycan of algal cells (Yin et al., 2012). Trypsase and pepsin were purified from *Bovine pancreas* and porcine gastric mucosa, respectively, and both enzymes can break down proteins of algal cell walls. Although these four enzymes are from other species, the conserved function of the same protein should be very similar. Our results approved that the enzymes showed a partial or obvious inhibition of the growth of algal cells (Figure 6). This suggested that four enzymes really functioned in breaking down the algal cells. Of course, some fungi produce antibiotics to inhibit the growth of cyanobacteria (Redhead and Wright, 1980), but other fungi can directly degrade algal cells (Jia et al., 2010; Han et al., 2011). Combining the enriched degradation activities related GO terms, enriched KEGG pathways, expression changes of all CAZyme genes, and inhibition effects of tested decomposition enzymes during algicidal process, we proposed that the algicidal mode of *T. versicolor* F21a might be associated with decomposition enzymes and metabolic pathways. It is known that antibiotics can inhibit the growth of bacteria. The secretion of antibiotics from fungi still can not be avoided by the present study. However, the breakdown of algae cells and reduced chlorophyll could not result from antibiotics within hours. Moreover, the main up-regulated decomposition genes mined here can guide the further isolation of efficient enzymes from *T. versicolor* F21a or the heterogeneous expression of decomposition enzyme(s) to inhibit algal bloom. Interestingly, it is a potential to use algal bloom materials as a medium for growing white rot fungi (Jia et al., 2012). The activities of several extracellular enzymes of *T. versicolor* F21a were significantly increased during the algicidal process (Du et al., 2015; Gao et al., 2017). Therefore, we hypothesized that the algal bloom materials can be used to produce inexpensive commercial decomposition enzymes in the future.

## Conclusions

In this study, ~66% of predicted fungal coding genes during the algicidal process could be detected via RNA-Seq. The total expression levels of all CAZyme genes during the algicidal process were increased by two folds compared with the control. Four tested homologous CAZymes such as cellulase, β-glucanase, trypsase, and pepsin were approved to partially inhibit the growth of algae. The algicidal mode of *T. versicolor* F21a might be associated with decomposition enzymes and metabolic pathways.

## Author contributions

WD, XC, and XW carried out majority of the experiment and bioinformatic studies. ZX and XG performed some experiments. WD, XC, and XW wrote the manuscript. CJ and RD helped draft the manuscript. GH conceived and directed the study. All authors read and approved the final manuscript.

### Conflict of interest statement

The authors declare that the research was conducted in the absence of any commercial or financial relationships that could be construed as a potential conflict of interest.

## Supplementary material

Supplementary Figure 1 Total RNAs extracted from mycelia co-cultivated with algal cells (treat) and pure mycelia (control) of 0, 6, 12, and 30 h samples. Rep1, replicate 1; rep2, replicate 2.

Supplementary Figure 2 Quality of raw reads of two arbitrarily selected samples.

Supplementary Figure 3 Comparison of expression changes between Real-time PCR and RNA-Seq.

Supplementary Figure 4 Number of members of lignocellulose families detected via transcriptomic and proteomic analysis. Total, number of all families; Detected by RNA-Seq, the number of expressed family genes detected by RNA-Seq; Diff by RNA-Seq, the number of differentially expressed family genes detected by RNA-Seq; Detected by Proteomics, the number of expressed family genes detected by previous proteomics study; Diff by Proteomics, number of differentially expressed family genes detected by the previous proteomics study.

Supplementary Figure 5 Expression changes of transcript (ID 37895) in the GH18 module.

Supplementary Table 1 Primers used for this study.

Supplementary Table 2 Statistics of RNA-Seq reads mapping results.

Supplementary Table 3 Numbers of up-regulated and down-regulated genes.

Supplementary Table 4 Expression levels of each CAZyme gene during the algicidal process.
