# Supplementary figures and images for: The Algicidal Fungus Trametes versicolor F21a Eliminating Blue Algae via Genes Encoding Degradation Enzymes and Metabolic Pathways Revealed by Transcriptomic Analysis
Source: Front Microbiol. 2018 Apr 27;9:826. doi: 10.3389/fmicb.2018.00826 (PMC5934417; doi:10.3389/fmicb.2018.00826)

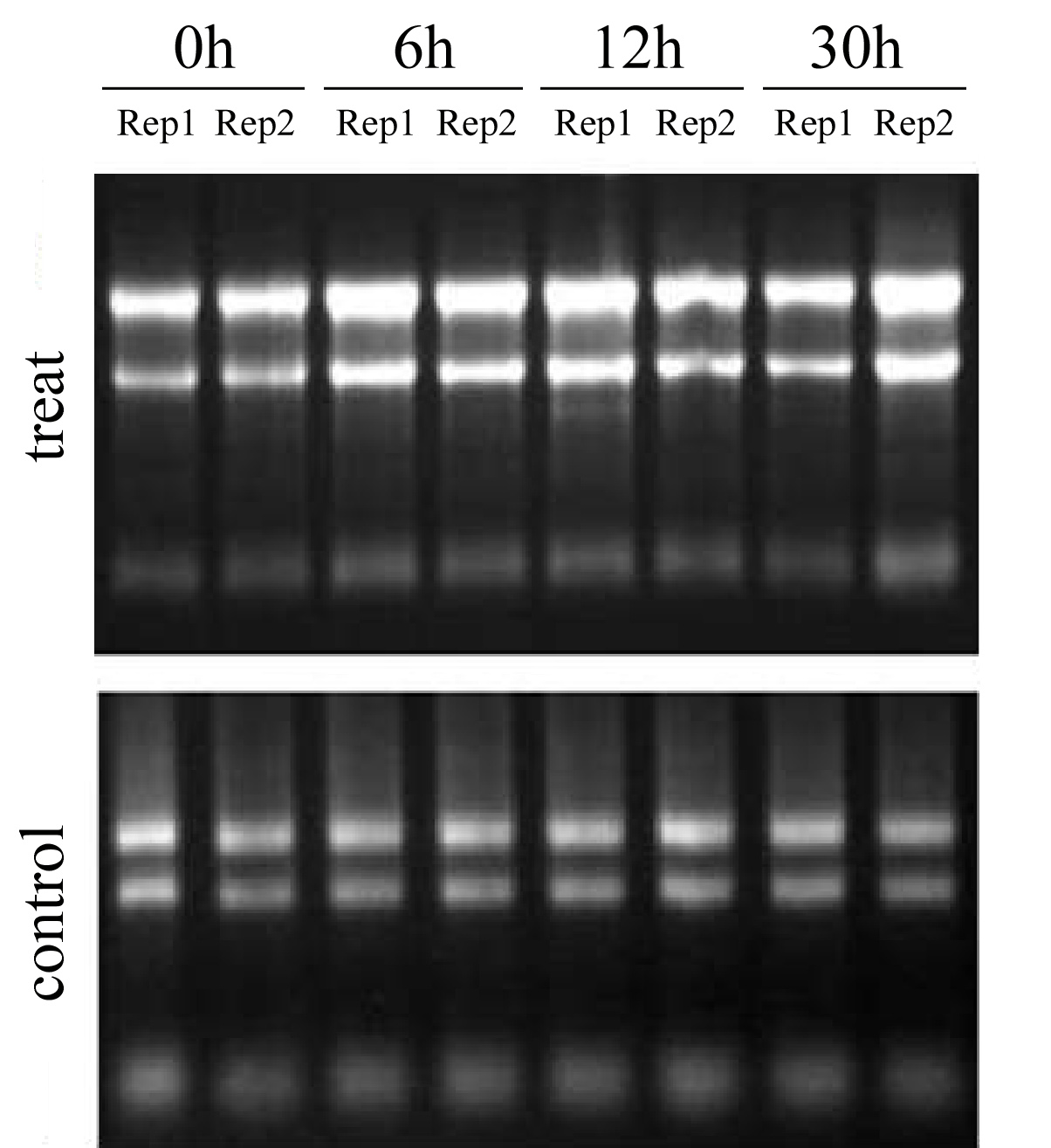

Supplement: Supplementary Figure 1 — Total RNAs extracted from mycelia co-cultivated with algal cells (treat) and pure mycelia (control) of 0, 6, 12, and 30 h samples. Rep1, replicate 1; rep2, replicate 2. [file Image_1.JPEG]

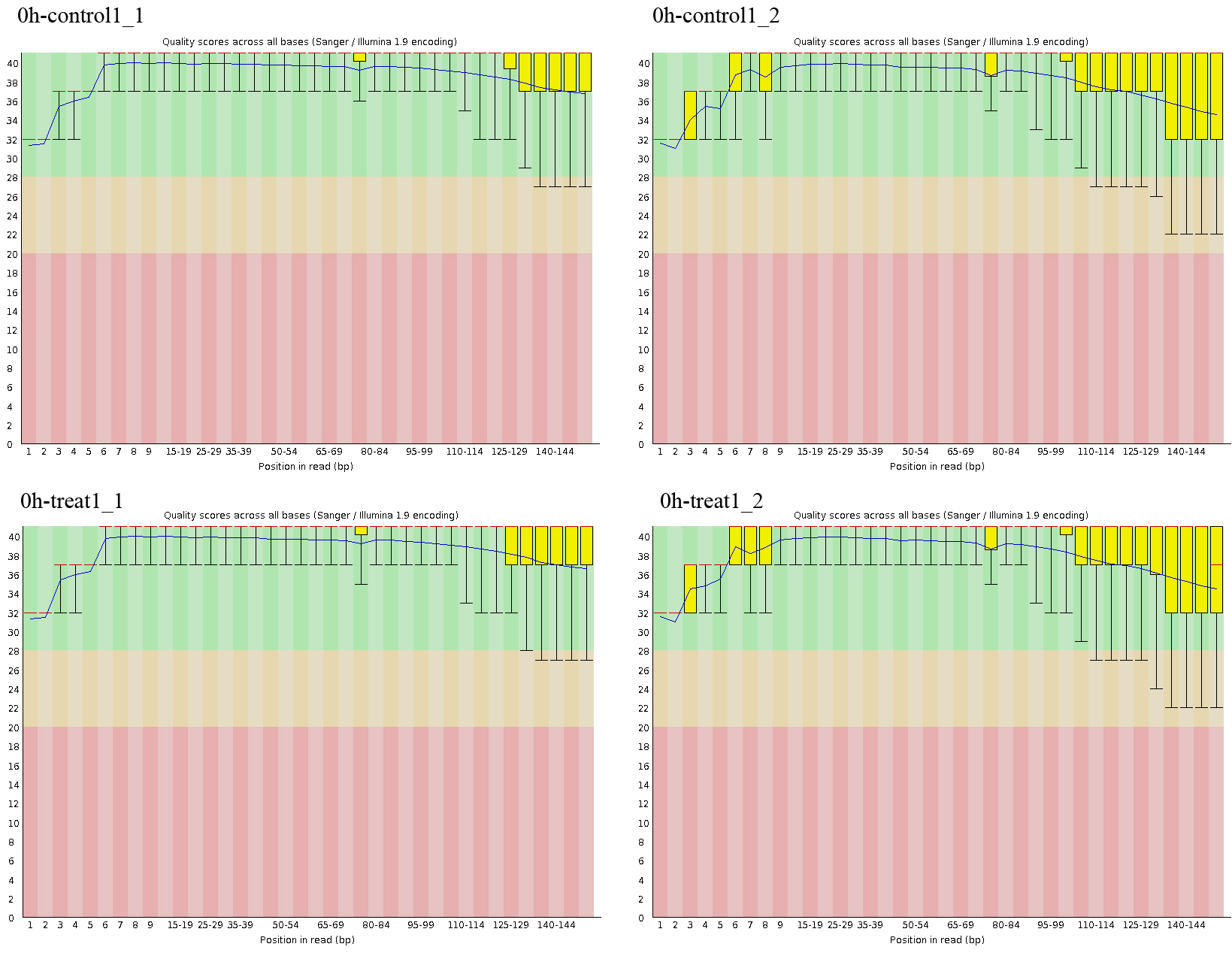

Supplement: Supplementary Figure 2 — Quality of raw reads of two arbitrarily selected samples. [file Image_2.JPEG]

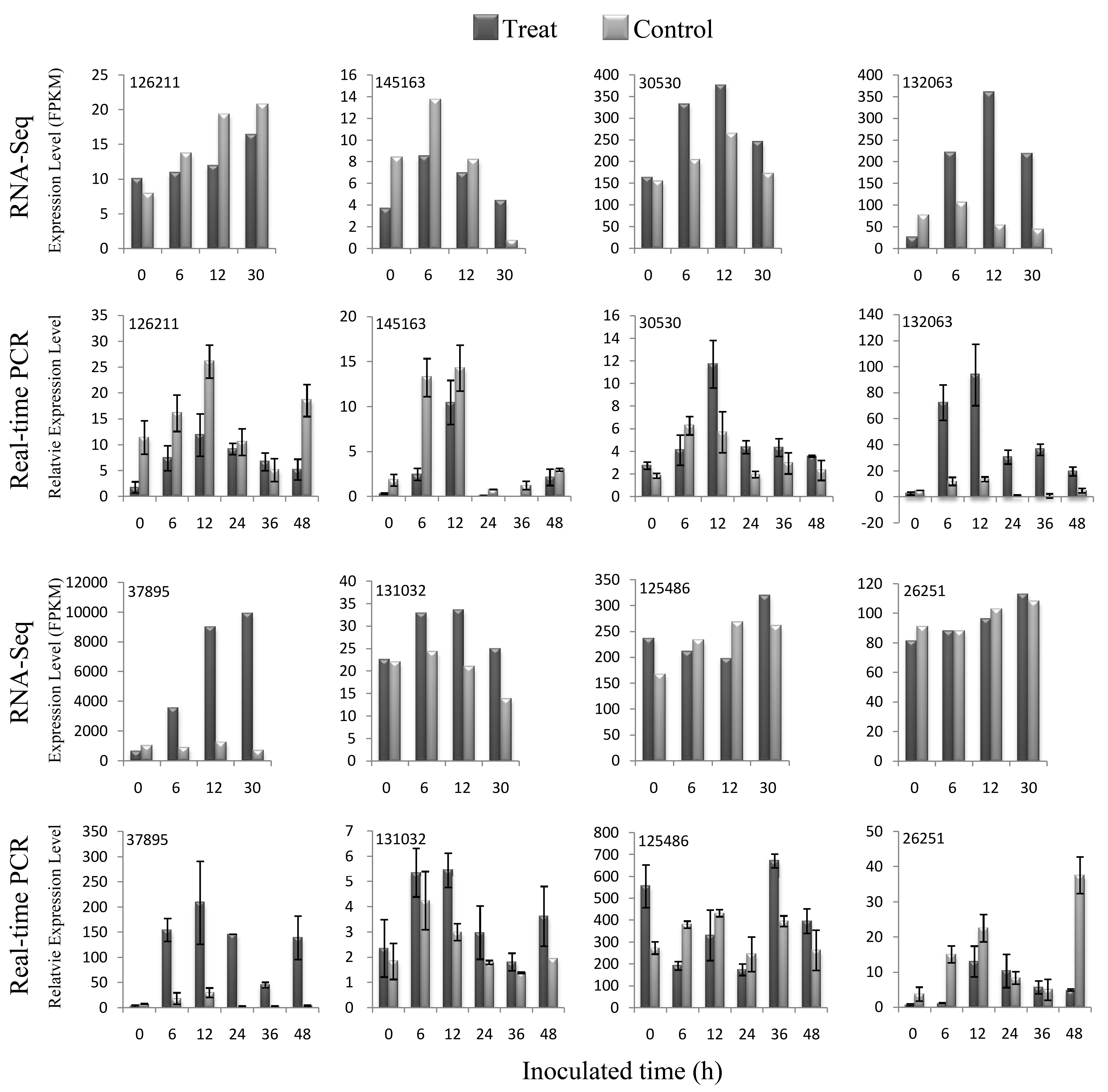

Supplement: Supplementary Figure 3 — Comparison of expression changes between Real-time PCR and RNA-Seq. [file Image_3.JPEG]

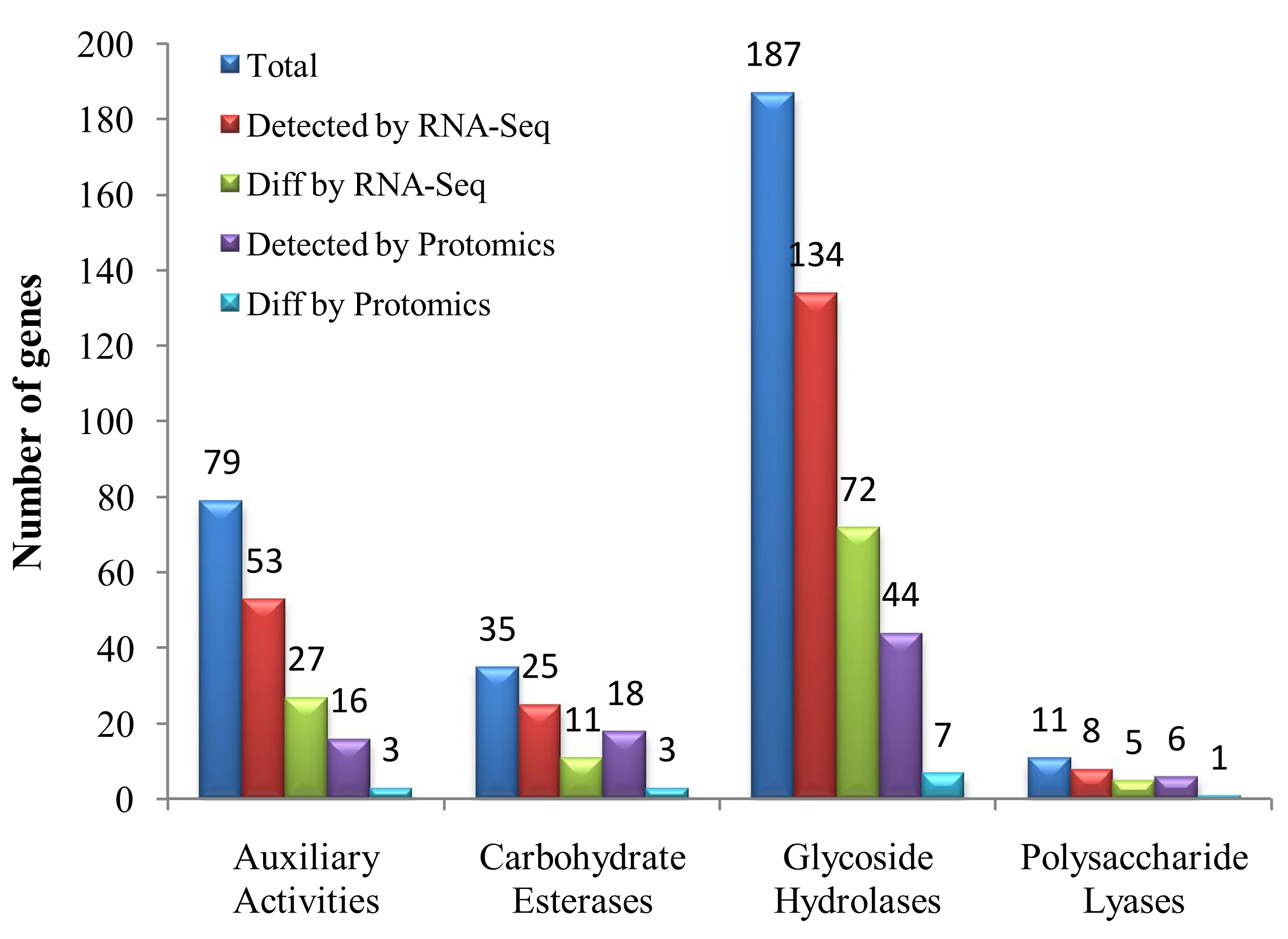

Supplement: Supplementary Figure 4 — Number of members of lignocellulose families detected via transcriptomic and proteomic analysis. Total, number of all families; Detected by RNA-Seq, the number of expressed family genes detected by RNA-Seq; Diff by RNA-Seq, the number of differentially expressed family genes detected by RNA-Seq; Detected by Proteomics, the number of expressed family genes detected by previous proteomics study; Diff by Proteomics, number of differentially expressed family genes detected by the previous proteomics study. [file Image_4.JPEG]

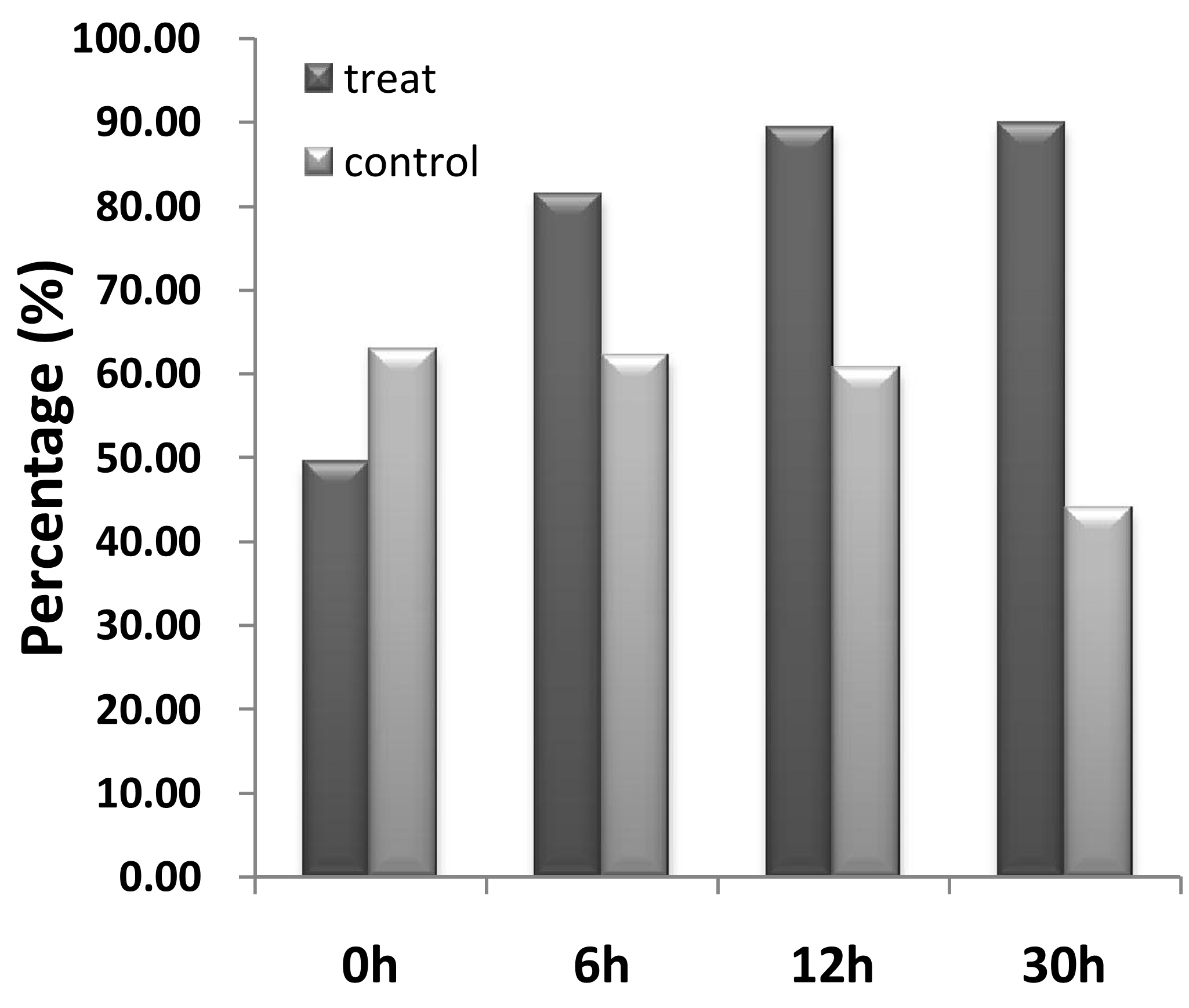

Supplement: Supplementary Figure 5 — Expression changes of transcript (ID 37895) in the GH18 module. [file Image_5.JPEG]
