# Supplementary material for: The Algicidal Fungus Trametes versicolor F21a Eliminating Blue Algae via Genes Encoding Degradation Enzymes and Metabolic Pathways Revealed by Transcriptomic Analysis
Source: Front Microbiol. 2018 Apr 27;9:826. doi: 10.3389/fmicb.2018.00826 (PMC5934417; doi:10.3389/fmicb.2018.00826)
Supplement: Supplementary Table 1 — Primers used for this study. [file Table_1.DOCX]

**Supplementary Table 1.** Primers used in this study.

| Coding sequence ID | Primer sequence |
| --- | --- |
| 126211 | CGGCTCGCGCGTGTAC |
|  | CTGGCCGTTGATGAACTTGA |
| 145163 | TGTACTCCTGGGATGTCGTGAA |
|  | GTACCAATACCGTCGATCAGCTT |
| 30530 | GTCGATGTCGTCGTCAACCA |
|  | CCACGCTGTAGTTGCTGACAA |
| 132063 | GCGTCCTCGCTGTTCCTATC |
|  | GCAGCGTATTCAGCCTTCTTG |
| 37895 | CGCCCTGATTGACTGGTACA |
|  | TTGCCGATGACGAGCTTGT |
| 131032 | TCCTGTCTACTTCGTGGTCATCAG |
|  | AGTATCCGCCGAATGTGTTGT |
| 26251 | CATCCCAGCCCTCGTCTTT |
|  | GCTGCCGCTACCATTGGT |
| 125486 | CGTGGACAGCCCGTATGG |
|  | GAACGTGCTCGGGTCGTT |
