# Supplementary material for: The Algicidal Fungus Trametes versicolor F21a Eliminating Blue Algae via Genes Encoding Degradation Enzymes and Metabolic Pathways Revealed by Transcriptomic Analysis
Source: Front Microbiol. 2018 Apr 27;9:826. doi: 10.3389/fmicb.2018.00826 (PMC5934417; doi:10.3389/fmicb.2018.00826)
Supplement: Supplementary Table 2 — Statistics of RNA-Seq reads mapping results. [file Table_2.DOCX]

**Supplementary Table** **2.** Statistics of RNA-Seq reads mapping results.

| Sample | Raw reads | Number of input reads | Cleanedlength | Uniquely mapped reads number | Uniquely mapped reads (%) | Average mapped length | Reads mapped to multiple loci(%) | Reads unmapped: too short(%) |
| --- | --- | --- | --- | --- | --- | --- | --- | --- |
| 0h-control1 | 4271398 | 4267416 | 143.99 | 2982950 | 69.90 | 273.85 | 3.04 | 27.06 |
| 0h-control2 | 4066994 | 4062652 | 143.58 | 2692948 | 66.29 | 272.97 | 3.11 | 30.61 |
| 0h-treat1 | 4218296 | 4213524 | 143.85 | 2857986 | 67.83 | 273.39 | 3.17 | 29.00 |
| 0h-treat2 | 4226619 | 4221749 | 143.82 | 2883284 | 68.30 | 273.38 | 3.07 | 28.63 |
| 6h-control1 | 4179240 | 4174787 | 143.87 | 2864909 | 68.62 | 273.6 | 2.85 | 28.52 |
| 6h-control2 | 4196724 | 4191856 | 143.79 | 2954030 | 70.47 | 274.24 | 2.94 | 26.58 |
| 6h-treat1 | 4203375 | 4199477 | 144.23 | 2844146 | 67.73 | 274.55 | 3.29 | 28.98 |
| 6h-treat2 | 4156707 | 4152046 | 144.02 | 2841402 | 68.43 | 274.32 | 3.07 | 28.50 |
| 12h-control1 | 3962286 | 3957410 | 143.40 | 2599471 | 65.69 | 272.43 | 2.82 | 31.49 |
| 12h-control2 | 4085467 | 4080528 | 143.73 | 2686274 | 65.83 | 273.19 | 2.83 | 31.33 |
| 12h-treat1 | 4236602 | 4231669 | 144.13 | 2887483 | 68.24 | 273.77 | 3.99 | 27.77 |
| 12h-treat2 | 4078243 | 4074062 | 143.57 | 2774321 | 68.10 | 272.58 | 2.95 | 28.95 |
| 30h-control1 | 4234905 | 4230179 | 144.08 | 2898644 | 68.52 | 273.66 | 2.97 | 28.50 |
| 30h-control2 | 4196589 | 4191810 | 143.86 | 2846319 | 67.90 | 273.05 | 3.00 | 29.10 |
| 30h-treat1 | 4117188 | 4112234 | 144.03 | 2722762 | 66.21 | 273.89 | 2.88 | 30.91 |
| 30h-treat2 | 3913014 | 3908163 | 143.69 | 2686514 | 68.74 | 272.92 | 2.95 | 28.31 |

Note: The number of reads were expressed in pairs.
