# Supplementary material for: The Algicidal Fungus Trametes versicolor F21a Eliminating Blue Algae via Genes Encoding Degradation Enzymes and Metabolic Pathways Revealed by Transcriptomic Analysis
Source: Front Microbiol. 2018 Apr 27;9:826. doi: 10.3389/fmicb.2018.00826 (PMC5934417; doi:10.3389/fmicb.2018.00826)
Supplement: Supplementary Table 3 — Numbers of up-regulated and down-regulated genes. [file Table_3.DOCX]

**Supplementary Table** **3.** Number of up-regulated and down-regulated genes.

| Different Samples | Up | Down | Total |
| --- | --- | --- | --- |
| 0h-treat/0h-control | 83 | 110 | 193 |
| 6h-treat/6h-control | 187 | 145 | 332 |
| 12h-treat/12h-control | 268 | 277 | 545 |
| 30h-treat/30h-control | 460 | 282 | 742 |
| 6h-treat/0h-treat | 490 | 601 | 1091 |
| 12h-treat/0h-treat | 514 | 524 | 1038 |
| 30h-treat/0h-treat | 597 | 422 | 1019 |
